# Temporal resolution in individuals with neurological disorders

**DOI:** 10.6061/clinics/2015(09)02

**Published:** 2015-09

**Authors:** Camila Maia Rabelo, Jeffrey A Weihing, Eliane Schochat

**Affiliations:** I Faculdade de Medicina da Universidade de São Paulo, Departamento de Fisioterapia, Fonoaudiologia e Terapia, São Paulo/SP, Brasil; II University of Louisville, Division of Communicative Disorders, Louisville/Kentucky, USA

**Keywords:** Temporal Lobe Epilepsy, Auditory Perception, Auditory Perceptual Disorder

## Abstract

**OBJECTIVE:**

Temporal processing refers to the ability of the central auditory nervous system to encode and detect subtle changes in acoustic signals. This study aims to investigate the temporal resolution ability of individuals with mesial temporal sclerosis and to determine the sensitivity and specificity of the gaps-in-noise test in identifying this type of lesion.

**METHOD:**

This prospective study investigated differences in temporal resolution between 30 individuals with normal hearing and without neurological lesions (G1) and 16 individuals with both normal hearing and mesial temporal sclerosis (G2). Test performances were compared, and the sensitivity and specificity were calculated.

**RESULTS:**

There was no difference in gap detection thresholds between the two groups, although G1 revealed better average thresholds than G2 did. The sensitivity and specificity of the gaps-in-noise test for neurological lesions were 68% and 98%, respectively.

**CONCLUSIONS:**

Temporal resolution ability is compromised in individuals with neurological lesions caused by mesial temporal sclerosis. The gaps-in-noise test was shown to be a sensitive and specific measure of central auditory dysfunction in these patients.

## INTRODUCTION

Temporal processing refers to the ability of the central auditory nervous system (CANS) to encode and detect subtle changes in acoustic signals, and normal temporal processing is necessary for perception of these acoustic changes 1-3. This processing has been further identified as being critical for normal auditory development 4. In the clinic, temporal processing can be evaluated behaviorally through measures such as the gaps-in-noise (GIN) test. This test was developed by Musiek in 2005 to characterize the ability of the CANS to resolve very brief acoustic changes. In particular, patients participating in the test are asked to press a button as quickly as possible as soon as a brief gap in a noise burst is identified 3.

The utility of the GIN test has been examined internationally in a variety of different populations 3,. In North America, normal-hearing individuals present gap detection thresholds (GDThs) of approximately 5 ms (4.8 ms for the left ear and 4.9 ms for the right ear) 3. Other researchers have also noted that GDThs of approximately 5 ms, with no significant differences between ears 7. Similar thresholds have been found in other normal-hearing 10 adults as in the previous studies 7. Examining non-neurological clinical groups, Sanches and colleagues found increased GDThs in individuals with tinnitus and normal peripheral hearing compared with normal-hearing individuals without tinnitus 10. This finding suggests that individuals with tinnitus may have alterations in their temporal resolution ability.

The GIN test has also been studied in patients with CANS involvement to characterize the temporal resolution ability of these individuals as well as to compute sensitivity and specificity because the GIN test is considered to be more sensitive for cortical lesions than other tests are 12. Findings have indicated that individuals with CANS lesions show deficits in their temporal resolution ability, as revealed by elevations in GDThs and decreases in the number of correctly identified gaps 3,5,13. Furthermore, clinical decision analysis has indicated that the test has good sensitivity and specificity, particularly for patients with cortical lesions 3,5,13.

One clinical group with neurological dysfunction of the CANS that has not been widely investigated with the GIN test comprises patients with mesial temporal sclerosis (MTS). MTS is the most common cause of temporal lobe epilepsy. All patients with MTS have neuronal loss and atrophy, but cortical dysplasia is present in only 15% of the cases. Certain studies have also shown a decreased gray-white matter demarcation in the temporal lobe parenchyma 14. As the temporal lobe is a region that is highly related to auditory processing, changes in this area may affect the auditory skills of these patients.

It is well known that MTS patients have problems with certain auditory abilities in daily life, such as speech perception acuity and speech discrimination; the GIN detection test has been found to be useful for assessing these difficulties 15-17.

The objectives of the present study were to first describe the temporal resolution ability of individuals with possible neurological CANS involvement caused by MTS and to then compute the sensitivity and specificity of the GIN test in this specific neurological population. We hypothesized that individuals with this disorder would show increased GDThs on the GIN test and that the test would reveal a good balance between sensitivity and specificity (i.e., good test efficiency).

## MATERIALS AND METHODS

### Participants

A total of 46 individuals were evaluated and divided into two groups: group 1 (G1; control group) was composed of 30 individuals with normal hearing sensitivity and without hearing disorders or a neurological disorder (mean age of 24.9 years; standard deviation (SD)=3.3), and the neurological group, or group 2 (G2), included 16 individuals with MTS in either the right lobe (N=14) or the left lobe (N=2 individuals) (mean age of 38.9 years; SD=9.3). All individuals showed normal results for a standard audiological evaluation, with pure-tone thresholds at the octave frequencies from 250 through 8000 Hz, equal to 20 dB HL or better; normal speech recognition test results; and normal tympanometry. None of the subjects in either group had auditory symptoms. Additionally, no significant air-bone gaps or interaural threshold differences greater than 10 dB were observed. The inclusion criteria for the different groups were as follows: subjects in G1 had negative neurological histories and performances within the normal limits on the dichotic digits and duration pattern tests 18-20, and subjects in G2 had a diagnosis of MTS confirmed by imaging exams (fMRI) of the temporal regions (excluding the brainstem) in the right or left hemisphere and performances below the normal limits on the dichotic digits and duration pattern tests.

### Procedures

The study design and consent form were approved by the University Ethics Committee for the Analysis of Research Projects (protocol number 1126/05). Subjects were submitted to the following procedures: a case history interview, pure-tone audiometry, speech recognition tests, tympanometry, acoustic reflex tests, and the GIN test 3. All testing was conducted by the staff of the Speech-Language Pathology and Audiology Laboratory of Investigation in Auditory Processing of the SLP and Audiology Program.

### Description of the GIN Test

The GIN test is a CD-based measure that can be administered in a typical audiology clinic. In the present study, the test was administered in a sound-proof booth using a GSI-61 audiometer and a Sony CD player. The test stimuli were presented at an intensity of 50 dB SL above the pure-tone average at 0.5, 1, and 2 kHz. The test was always presented monaurally, with both ears being tested separately. The CD features one practice list and four test lists; each subject completed the practice list before beginning the experiment. A different test list was presented to each ear during the experiment, with one in the right ear and another in the left ear.

In the GIN test, subjects identify when gaps occur in a noise stimulus. The noise is a Gaussian-distributed white noise generated with a sampling rate of 44.1 kHz. Noise segments are 6 s in duration, within which 0 to 3 gaps may be embedded. The gap durations are 2, 3, 4, 5, 6, 8, 10, 12, 15, and 20 ms. In each GIN list, the individual gap durations are presented 6 times each in random locations across the various trials, for a total of 60 gaps. Moreover, certain noise trials do not contain any gaps. The shortest time interval between gaps in a noise burst is 500 ms, and the longest interval is on the order of seconds. Each trial is 6 s in duration, during which the noise stimulus is presented, and the interval between trials is 5 s. The four different GIN lists have been shown to be equivalent 3,8.

In the present study, subjects were instructed to press the response button as soon as they heard a gap. If a gap occurred and the response button was not pressed, this was counted as an error. If no gap occurred but the button was pressed, it was counted as a false positive. The examiner clarified any confusion regarding the responses by asking the subject how many gaps were detected in that specific segment to confirm the number of correct responses. The approximate GDTh for each test list was defined as the shortest gap perceived by the subject at least 66.6% of the time, which was four times per list. If a subject's performance fluctuated by gap duration (e.g., 4 of 6 correct at 4 ms, 2 of 6 correct at 5 ms, 4 of 6 correct at 6 ms), the gap duration at which the performance stopped fluctuating was considered as the GDTh (6 ms in the preceding example). Normal performance on this test was defined by Samelli and Schochat 7 as a GDTh of 4.19 ms and a correct identification score of 78.5% or greater.

## RESULTS

With respect to the age of the individuals, G2 (the neurological group) showed a higher average age than G1 (the control group) did (averages: G1=25 years old and G2=38 years old).

Table 1 shows the descriptive statistics for both groups' GIN testing. The results are presented as the mean GDTh (in ms) and the percentage of correctly identified gaps. The mean threshold obtained for G1 was significantly smaller than that for G2 (*p*<0.001), indicating better gap detection performance in individuals without MTS. In both groups, however, no significant difference was detected between the left and the right ears (G1: *p*=0.227; G2: *p*=0.432). Additionally, the mean percentage of correct data (Table 2) indicated that G2 scored significantly more poorly than G1 did. Finally, for the MTS group, a comparison of performance between the ear ipsilateral to the lesion and the ear contralateral to the lesion did not reveal a significant difference in the GDTh (*p*=0.564) or the percentage of correct identification (*p*>0.999) (Table 3).

**Figure 1:**
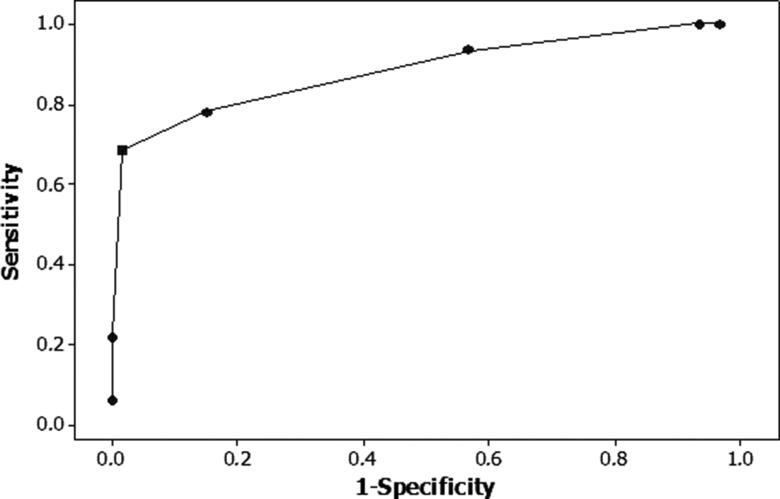
Receiver operating characteristic curves for the gaps-in-noise test threshold, comparing the normal and neurological groups.

**Figure 2:**
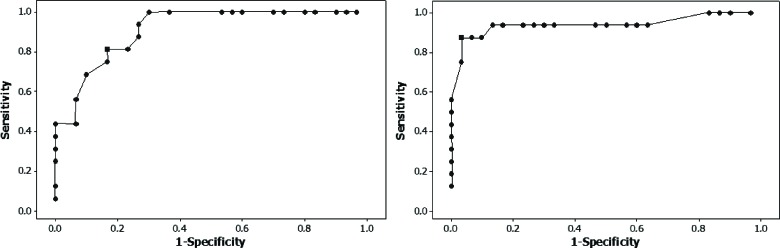
Receiver operating characteristic curves for the percentage of correct answers in the gaps-in-noise test, comparing the right and left ears in the normal and neurological groups.

As stated previously, a second purpose of the present study was to establish the sensitivity and specificity of the GIN test in patients with MTS. To this end, receiver operating characteristic (ROC) curves were created to characterize the relationship between sensitivity and specificity for a variety of different cut-off criteria. The optimal criteria were assumed to be those that yielded the best balance between sensitivity and specificity. Because no significant difference was found between ears by comparing the thresholds, the ears were grouped and averaged in the creation ROC curves (Figure 1). Figure 2 shows the ROC curves for the percentage of correct answers for the right and left ears. The most efficient cut-off was 6.8 ms (e.g., ≥8 ms GDTh on the GIN), at which the sensitivity was 68% and the specificity was 98%. For the percentage of correct responses, for the right ear, the most efficient cut-off was 70.6%, at which the sensitivity was 85% and the specificity was 73%. For the left ear, the most efficient cut-off was 71%, at which the sensitivity was 78% and the specificity was 73%.

## DISCUSSION

The major results of the present study can be described as follows: patients with MTS had significantly increased GDTh values and lower percentages of correct gap detection than neurologically normal controls did, indicating significant impairments in temporal processing in this clinical population; a GDTh criterion of ≥8 ms yielded the best balance of sensitivity and specificity for MTS lesions (68% and 98%, respectively); a percentage of correct identification of 70% yielded the best balance between sensitivity and specificity (approximately 80% and 73%, respectively); and no significant difference was found in the GDTh or the percentage of correct responses between ears in either group.

### Comparison with Previous Literature on Normal-Hearing and Neurological Populations

Regarding age, individuals in G2 were older than those in G1 (averages: G1=25 years old and G2=38 years old). However, recent studies on normal individuals from 21 to 45 years of age showed that there is no correlation between adult subjects' age and GIN test thresholds; in fact, the GDTh was consistently 4.7 ms, suggesting that age does not affect the GDTh in adults 10,21. Another study found an adult GDTh of 5.43 ms (established by adding 2 SDs to the mean GDTh) 7.

The findings of the present study are in good agreement with results from previous studies investigating GIN test performance in normal-hearing populations. Specifically, normal-hearing listeners in one prior study 3 showed GDTh values that were very similar to those noted in the present study (∼4.8 ms in their findings and ∼4.7 ms for the left ear and ∼4.6 ms for the right ear in the present study) (Table 1). Similarly, two other studies also noted mean GDTh values in normal-hearing listeners 6,21, or 5 ms and 4.7 ms, that were similar to those obtained in the present study. The present findings further suggested that the ear did not affect GIN test performance in the normal-hearing population, corroborating a result of Samelli and Schochat 8. Regarding the percentage of correct responses, in the current study, G1 also showed values that were similar to those reported in other studies 3,8. In fact, only one study in the literature 7 is incongruent with the results for normal listeners in the present study; that study reported a GDTh value (4.19 ms) smaller than that found in the present sample, although this difference was not clinically meaningful. However, if we add the SD to the mean, the obtained value is similar to the value suggested by the study with normal criteria (5.43 ms).

Findings for subjects with confirmed CANS involvement in the present study were also generally consistent with previous studies. In particular, values similar to those obtained in the current study were reported in the literature 3, in which the GDThs obtained for individuals with CANS disorders were 8.5 ms for the right ear and 7.8 ms for the left ear. Furthermore, other research 5 noted that neurological subjects had abnormal GDThs in both ears. The authors specifically administered the GIN test to 8 individuals with neurological dysfunction and noted abnormal performance in all cases. Thresholds were bilaterally abnormal in five cases (four with a right lesion and one with a left lesion) and abnormal only in the ear contralateral to the lesion in three cases (two with a left lesion and one with a right lesion). All of the thresholds were between 6 ms (SD=2) and 9 ms (SD=1) for the left ear and between 8 ms (SD=2) and 11 ms (SD=3) for the right ear.

As found in our study, others researchers 22 showed worse results in neurological patients. These researchers studied gap detection in individuals with acquired aphasia secondary to left hemisphere lesions using two different mechanisms, or “within-channel” and “between-channel” conditions. The results showed that individuals with aphasia averaged fewer correct responses than age-matched neurologically intact controls did and that as the gap duration increased, the mean number of correct responses increased in both the control and the aphasic subjects. The results also suggested that left hemisphere lesions prevent perceptual discriminations that require the precise analysis of information that unfolds or rapidly changes over time.

Based on these results, we can say that MTS patients represent a clinical population that may clearly have an auditory deficit. Audiologists also may see these patients and should be aware that these patients could have central auditory deficits. Audiologists should also be aware that a normal pure-tone audiogram in patients with MTS may not reveal the whole story.

### Lack of Laterality in the Present Findings

Because our study group was small (only 14 individuals with right MTS and 2 with left MTS), it is difficult to affirm that the side of the lesion influences GIN test performance.

Although a numerical trend indicated that the ear contralateral to the lesion scored more poorly than the ear ipsilateral to the lesion did, this difference did not reach statistical significance (Table 3). This finding is somewhat consistent with previous research, which has also indicated that no significant laterality effects exist in neurological populations. For instance, poorer GDTh values are not always observed in the ear contralateral to the injured hemisphere 23. Furthermore, another study 3 noted that only four of eighteen individuals in a sample of neurological patients demonstrated a contralateral ear effect, and no significant within-group differences were found for the left ear compared with the right ear for the GDTh or for the percentage of correct responses for either group, with or without lesions. Additionally, in 2006, researchers found an increased GIN test threshold for the ear contralateral to the lesion in only three cases and bilaterally in five cases 5.

The results of a recent study revealed that no significant differences existed between the right and the left ears in either normal individuals or MTS patients. According to the authors, this finding could suggest that MTS makes the central nervous system vulnerable to temporal processing deficits 12. Contralateral seizure propagation in patients with mesial temporal lobe epilepsy has also been reported in the literature, indicating that the condition may affect both sides 24.

These results are probably related to the fact that the response to the GIN test involves the whole cortex, and not only its contralateral pathways 3.

### Clinical Decision Analysis

Sensitivity and specificity indices were calculated based on the performance of the subjects in the present study (Figures 1 and 2). ROC curves indicated that a 6.8 ms cut-off for the GDTh and a 68% cut-off for the percentage of correct responses yielded the best overall balance between diagnostic indices. At these cut-offs, a sensitivity and a specificity of 68% and 98%, respectively, were noted for the GDTh, whereas a sensitivity and a specificity of 81% and 83%, respectively, were noted for the percentage of correct responses. These findings indicate that the GIN test shows good efficiency in detecting lesions of the mesial temporal lobe.

The hit rates for central auditory dysfunction in the present study are somewhat consistent with what has previously been noted. Previous results specifically showed a sensitivity of 67% and a specificity of 94% for a GDTh of 7 ms 3. Conversely, the ROC curves for the percentage of correct responses were slightly different between the present study and the previous results in the literature 3. The differences between the studies may have been influenced by the differences in age and lesion sites between the study samples. More specifically, the mean age in Musiek's study 3 was higher than that in the current study, and that study's subjects had more extensive lesions than in the present study; the lesions ranged from the brainstem to the right and left hemispheres, rather than being restricted to the auditory cortex.

### Auditory Contributions from the Mesial Temporal Lobe

The regions affected by MTS can be relatively diffuse. In patients with this condition, several authors have noted that abnormalities are associated with one or more of the following regions: the hippocampus, temporal neocortex, insula, uncus, precentral gyrus, thalamus, parietal lobe, cuneus, and cingulum. Individuals with severe seizure conditions also show abnormalities in the temporal and extratemporal lobes 25,26. Furthermore, fMRI data have indicated that MTS decreases the functional connectivity between auditory regions and other sensory areas of the cerebrum such as somatosensory areas 27.

Despite the diffuse nature of these lesions, multiple studies, including the present investigation, have noted poor performance on auditory measures in patients with MTS. In 2011, one study showed that individuals with MTS exhibited significantly elevated auditory electrophysiological thresholds, as measured based on the auditory steady-state response compared with controls, even though the two groups had similar behavioral thresholds 28. This result suggests that the neural function of MTS patients does not contribute the neural synchrony necessary for the detection of subtle electrophysiological events. This finding was further supported by studies showing electrophysiologic abnormalities in event-related potentials (ERPs) recorded from patients with MTS 29, which were not observed using a similar paradigm in the visual modality 30.

The current study extends this area of knowledge to suggest that in addition to having dysfunction in the central auditory system, functional difficulties are observed in the encoding of rapid changes in the fine structure of the auditory signal in patients with MTS. This finding supports previous research suggesting reduced gap detection ability and poor temporal processing in patients with neurological dysfunction of the central auditory system 3,5.

### Clinical Implications

The present study supports the utility of the GIN test in patients with MTS. Furthermore, the findings presented here support previous investigations indicating that the GIN test, and gap detection ability more generally, is a sensitive and specific measure of CANS dysfunction. The lack of an ear effect in the present study suggests the possibility of testing a single ear or administering the GIN test bilaterally when using the measure clinically. However, the latter approach would require validation in a neurological population before widespread clinical use.

## Figures and Tables

**Table 1:** Descriptive statistics for the gap detection threshold (ms) in the gaps-in-noise test for both groups by ear.

	N	Mean	Standard Deviation	Median
**Group RE**				
G1 – Normal	30	4.7	1.0	5
G2 – MTS	16	7.4	2.9	7
**Group LE**				
G1 – Normal	30	4.6	1.0	5
G2 – MTS	16	8.1	1.7	8

Legend: RE = right ear; LE = left ear; N = total number of subjects; G1 = group 1; G2 = group 2; MTS = mesial temporal sclerosis

**Table 2:** Descriptive statistics for the percentage of correct answers in the gaps-in-noise test for both groups by ear.

	N	Mean	Standard Deviation	Median
**Group RE**				
G1 – Normal	30	75.6	7.6	75
G2 – MTS	16	57.6	13	63.3
**Group LE**				
G1 – Normal	30	76.1	7.6	76.6
G2 – MTS	16	52.7	13.1	55.8

Legend: RE = right ear; LE = left ear; N = total number of subjects; G1 = group 1; G2 = group 2; MTS = mesial temporal sclerosis

**Table 3:** Descriptive statistics for the thresholds and the percentage of correct answers in the gaps-in-noise test for the ipsilateral and contralateral ears.

Ear	N	Mean	Standard Deviation	Median
Ipsi (threshold)	16	7.5	2.9	8
Contra (threshold)	16	8.0	1.8	8
Ipsi (%)	16	56.7	12.7	59.2
Contra (%)	16	53.6	13.7	57.5
